# Rare Presentation of Pulmonary Embolism Amidst Coronavirus Disease 2019 Era: Utility of Multiorgan Ultrasonography

**DOI:** 10.7759/cureus.8452

**Published:** 2020-06-05

**Authors:** Taha Ahmed, Talha Ahmed, Sany Kumar, Samra Haroon Lodhi, Bassel Akbik

**Affiliations:** 1 Internal Medicine, Cleveland Clinic Foundation, Cleveland, USA; 2 Internal Medicine, University of Maryland Medical Center, Baltimore, USA; 3 Internal Medicine, Cleveland Clinic - Fairview Hospital, Cleveland, USA; 4 Internal Medicine, King Edward Medical University, Lahore, PAK; 5 Internal Medicine, Mayo Hospital, Lahore, PAK; 6 Critical Care Medicine, Cleveland Clinic, Cleveland, USA

**Keywords:** coronavirus disease, covid-19, severe acute respiratory syndrome-coronavirus-2, sars-cov-2, multi-organ ultrasound, mou, chest computed tomogram, chest tomography angiogram, pulmonary embolism

## Abstract

Coronavirus disease 2019 (COVID-19) pandemic caused by severe acute respiratory syndrome-coronavirus-2 (SARS-CoV-2) is the underlying cause of a global crisis that the entire world is facing. It is a highly contagious viral infection, which is why social distancing seems to be effective. Its ability to survive on various surfaces and aerosolize necessitates very meticulous precautions, including airborne isolation for severely ill patients requiring mechanical ventilation. However, these patients may need routine diagnostic investigations including chest computed tomography and chest tomography angiogram scans (CT and CTA) to rule out other potential differential diagnoses and guide management. In this case, we focus on the utility of multiorgan ultrasonography (MOU) at the bedside to diagnose and manage pulmonary embolism (PE) in COVID-19 patients.

## Introduction

There has been a dramatic increase in the cases as well as mortality related to the coronavirus disease 2019 also known as COVID-19 ever since being first reported [1]. The cause of this current global crisis is a special strain of the coronavirus family. Primarily affecting the lung parenchyma, however, its manifestations on the human body are protean and not completely known. Rare cases of its correlation with pulmonary embolism (PE), presumed to be due to a virus-related inflammatory and prothrombotic state have been reported [2]. We describe a case of a patient with COVID-19 infection who developed acute PE that was diagnosed with multiorgan ultrasonography (MOU). The utility of MOU in limiting exposure to COVID-19 in healthcare settings as well as guiding the management of acute PE in these patients is discussed.

## Case presentation

A 74-year-old Caucasian male presented to the ED with worsening shortness of breath and elevated blood pressure. On presentation, he was markedly tachypneic and in severe respiratory distress. Vital signs include an elevated blood pressure of 165/114 mmHg, a pulse of 122 beats per minute, a temperature of 99.1°F and a respiratory rate of 28/minute. On physical examination, the patient was in acute distress, diaphoretic, and using accessory muscles of respiration. Lung auscultation revealed diffuse rales. Regular rate and rhythm with no accessory heart sounds were appreciated on precordial auscultation. The patient was put on a non-rebreather mask with mild improvement in the respiratory status and subsequently intubated and transferred to the intensive care unit on mechanical ventilation.

The patient’s medical history was significant for obstructive sleep apnea with noncompliance to positive airway pressure therapy due to discomfort. He had grade 1 left ventricular diastolic dysfunction on an echocardiogram 8 months ago with normal biventricular size and function. He took a trip to Florida with his wife and noticed body aches, constant non-productive cough, headaches, fatigue, and fevers with a temperature of 101°F since his return 10 days prior. He reported sick contact at his vacation, which prompted him to get checked for viral pathogens and get a chest x-ray. The patient was diagnosed with COVID-19 8 days prior to presentation and was followed by the Cleveland Clinic’s distance health service with daily phone encounters while he was in isolation at home. His chest x-ray had shown left perihilar opacity and he was provided with doxycycline.

The differential diagnosis included viral pneumonia, bacterial pneumonia, PE, and COVID-19 acute respiratory distress syndrome (ARDS).

**Figure 1:**
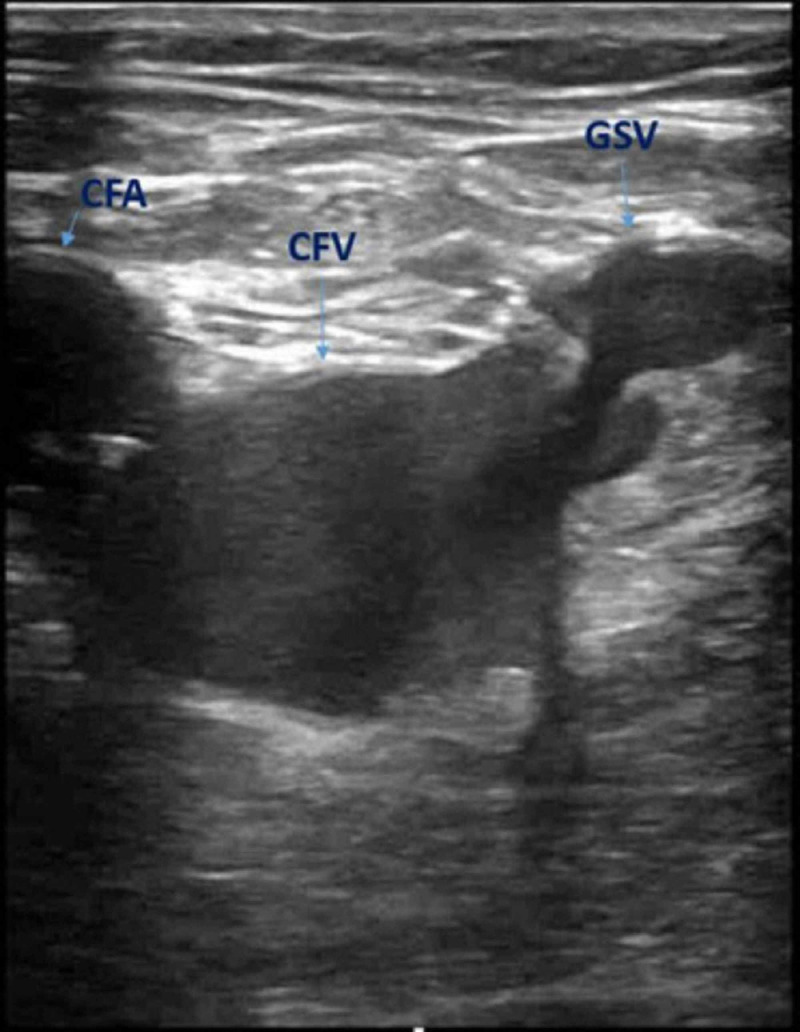
Point of care ultrasonography of the left lower extremity at the level of inguinal ligament in transverse view showing a clot in the common femoral vein and great saphenous vein In the transverse view, the great saphenous vein (GSV), common femoral vein (CFV) and the common femoral artery (CFA) create a recognizable image referred to as the "Mickey Mouse' Sign": GSV = ear; CFV = face; CFA = ear

Laboratory work revealed hemoglobin of 17.3 g/dL, white blood cell count of 16,400/uL, elevated liver enzymes with ALT 336 U/L and AST 309U/L. C-reactive protein was elevated to 11.9 mg/dL, D-dimer elevated to 4,300 ng/mL, IL-6 was elevated to 52 pg/mL and ferritin was elevated to 5440 ng/mL. Electrocardiogram (EKG) was insignificant for any acute abnormality, troponin T was elevated to 0.0770 ng/mL and proBNP was elevated to 1312 pg/mL. Arterial blood gas analysis revealed acute hypoxemic respiratory failure with a P: F of 220 on 80% fractional inspired oxygen (FiO2). Chest x-ray revealed bilateral hazy opacities. The patient was kept in airborne precautions. Blood and urine cultures were drawn. The tracheal aspirate was sent for bacterial pneumonia workup. A point of care lower extremity ultrasound revealed left femoral deep venous thrombosis (Figure 1).

Low tidal volume lung-protective ventilation was maintained with a high positive end-expiratory pressure (PEEP) and a low fraction of inspired oxygen (FiO2) strategy. The patient was initiated on hydroxychloroquine and azithromycin based on anecdotal data. The patient was a candidate for tocilizumab therapy but was avoided in the setting of abnormal liver function. His P: F ratio suddenly started to decrease along with the elevation of troponin T and pro-BNP. Suspicion of myocarditis, as well as a PE, were high on the differential. As multimodality imaging was limited due to strict precautions to prevent spread, transthoracic echocardiography was performed revealing a normal left ventricular size and systolic function but with a moderately dilated right ventricle with impaired right ventricular (RV) systolic function, severe hypokinesis of basal and mid RV walls and septum with normal contractility of distal RV wall and apex (Video 1).

**Video 1:** Transthoracic echocardiography in the apical four-chamber view showing impaired right ventricular (RV) systolic function, severe hypokinesis of basal and mid RV walls and septum with normal contractility of distal RV wall and apex

The pattern of RV wall motion was consistent with acute RV strain and a positive McConnell’s sign (Video 2).

**Video 2:** Acute right ventricular strain and positive McConnell's sign in apical two-chamber view

The patient was started on a heparin infusion. The patient’s acute inflammatory markers trended down with medical therapy and his ARDS improved. Troponin T also returned to baseline after 48 hours.

Hospital course was complicated by the development of ventilator-associated pneumonia and after being on the ventilator for 14 days. the patient was successfully extubated. The patient is currently recovering on the medical floor.

## Discussion

The pandemic caused by the novel strain of coronavirus, SARS-CoV-2, is one of the major challenges that the entire world is currently facing due to limited treatment options. After the first cases of SARS-CoV-2-related disease also known as COVID-19 were detected in Wuhan, China, the spread throughout the world has been extremely dramatic [3]. On January 30, 2020, the World Health Organization (WHO) declared the disease caused by the novel coronavirus (COVID-19) a public health emergency of international concern (PHEIC), later officially upgrading it as a global pandemic.

The clinical presentations are protean. In 88% of cases, fever is the most common presentation, followed by cough (68%), vomiting (5%), and diarrhea (3.8%). ARDS, multi-organ failure including acute kidney injury, disseminated intravascular coagulation are some of the dreaded complications in 15% of these patients often being the cause of death [4]. The presence of strict airborne isolation limits the utilization of computed tomography angiogram scans in these patients. There has been a wide institutional variation based on the number of suspected and diagnosed cases of COVID-19 as well as CT-scan machines available in a given hospital. In addition to this, the presence of acute renal injury or contrast allergy can also preclude the use of this gold standard modality in diagnosing PE in these patients [5]. Patients with COVID-19 have increased risk of venous thromboembolism and disseminated intravascular coagulation has been well described in studies from China and Italy.

For patients with intermediate probability of PE based on Well’s criteria, negative D-dimer testing can rule out a PE. COVID-19, however, being an inflammatory state can cause an elevation of D-dimer; hence most of these low-risk patients may end up needing a CTA for PE evaluation. For these patients, as well as for patients with high Well’s score, we describe the MOU technique as a good alternative to diagnosing PE in individuals with COVID-19 ARDS [6]. This can limit the exposure in healthcare settings to this extremely contagious disease. It is also a reasonable alternative for patients with contraindication to contrast use. An isolated echocardiogram or ultrasound of lower extremities may not achieve the same degree of sensitivity, but when combined they are 88% sensitive and 90% specific when compared to a CT angiogram [7].

Our patient who was initially diagnosed with COVID-19 and was being followed remotely under quarantine suddenly developed worsening shortness of breath and presented to the hospital. The underlying etiology of his worsening respiratory status was a likely sudden exacerbation of his viral illness and also contributed by acute PE. The patient underwent an MOU of the bilateral lower extremities as well as an echocardiogram. This not only helps in diagnosing deep venous thrombosis as the cause of his acute PE but also helped to quantify the severity using risk calculators. BOVA score, which predicts the PE-related complications in acutely sick patients, was high in our patient (BOVA score of 5). This suggests a high risk (42 %) of 30-day death from PE-related complications like hemodynamic collapse and recurrent PE [8].

Traditionally, echocardiography is neither sensitive nor specific for the diagnosis of PE. However, in patients with COVID-19, it remains an essential tool to look for signs of PE. It is an easy and quick method and useful to predict mortality in critically ill patients. Moderate or severe RV hypokinesis, persistent pulmonary hypertension, a patent foramen ovale, and free-floating right-heart thrombus are echocardiographic markers that identify patients at risk for death or recurrent thromboembolism. McConnell’s sign with a free-floating RV clot in our patient suggested a massive PE [9]. Fortunately, our patient’s hemodynamics including blood pressure stayed stable. He was carefully monitored in the intensive care unit with plans for further management including thrombolysis or thrombectomy in case he developed signs of hemodynamic instability [10,11]. With therapeutic heparin along with ventilator support for COVID-19, the patient gradually improved and his inflammatory markers trended down and was ultimately extubated. Heparin was transitioned to direct oral anticoagulant (DOAC) rivaroxaban.

## Conclusions

Amidst the current COVID-19 crisis, minimizing exposure in all settings including healthcare is the cornerstone of disease mitigation. We describe a case of acute PE in a known case of COVID-19 infection. The correlation of viral infection and PE remains unclear due to the paucity of current literature. An MOU can help in diagnosing the source and severity of PE and help us decide appropriate management. It is important that healthcare workers are able to understand venous thromboembolism as an important differential diagnosis in patients with COVID-19, particularly in critically ill patients. We also focus on the ability to utilize MOU as a good alternative to gold standard CTA in diagnosing PE, in order to minimize exposure in healthcare settings, to conserve essential resources in resource-limited hospitals, and in patients in whom contrast use is prohibitive.
